# Caries and Necrosis

**Published:** 1871-05

**Authors:** 


### ARTICLE VI.

### *Caries and Necrosis.*

The four cases sitting before you are all instances of a destructive inflammation of the maxillary bones, resulting in the death of a portion of their substance—they are instances of caries and necrosis. Now what is necrosis? Caries in bone is analagous to ulceration in tissues; necrosis, to mortification. One is degenerating, slight in degree and limited in extent; the other extensive and complete. They are both the result of inflammation, an ostitis which may be provoked in various ways, but is very commonly, in the maxillary bones, the result of the periodontitis. Ostitis does not, of course, always end in caries and necrosis, any more than does inflammation in the soft parts end in ulceration, yet in certain conditions, especially in struma, such a result may occur.

Caries of the maxillary bones, especially of the lower, is comparitively rare; yet these cases will occasionally come to our notice. I have spoken to you of alveolar abscess as a cause, and you see how often I bring up this subject in my lectures, to impress it more forcibly upon your minds, that it is a disease which is extremely common. Look at this skull, which I hold in my hand; all along this alveolar process, just against the extremities of the fangs of the teeth, you will see little openings, which appear as though the bone had been perforated ; and so it has, but not by an instrument. These openings are all the result of alveolar abscesses, and I have no doubt that many of you have precisely similar ones in your jaws.

Remember, then, to pay particular attention to these periodontal troubles, and do not let your patients suffer from either caries or necrosis, with their accompanying sinus-riddled faces, the scars of which will ever remind you of your ignorance or culpable neglect.

Caries may arise occasionally directly from ostitis, the result of injury, but periodontitis is much more commonly the first recognizable symptom as well as the cause, and should be combatted in its acute stage, as shown in my previous lecture—Reporter July 30th, 1870,—since, by a failure to thus vigorously treat it, I have seen extreme complications occur.

But suppose, as often happens, that you have not been called to the case until all this mischief has been done; you will then find the patient somewhat like this little girl:

*Case* I.—Who, as you will see the orifice of a sinus discharging pus, at the base of her jaw. The parts about, are red, indurated and excoriated, and she tells me that she has been in this condition for months. Her trouble, she informs me, and as I anticipated, commenced in a tooth ache, and this sinus soon followed. I pass the probe, and what do I feel? Bone, not smooth and covered with periosteum, but soft, riddled, easily broken down—very much like old honeycomb. Now, I know that this is not now simply an uncomplicated alveolar abscess. I am sure that in its progress the inflammation extended to the bone structure aud caries has been the result.

This little girl is pale and delicate, presenting those general appearances which we call strumous, and this circumstance has unoubtedly been largely instrumental in bringing about this unfortunate state of things. When will the day come, gentlemen, when you and I shall be able to speak intelligently of this taint "scrofula;" to define its meaning—to discover its cause—to trace its course—and best of all to grapple with and overpower it. I cannot stop here to discuss the question as to the consanguinity of the conditions, syphilis, scrofula, tubercle, etc., but much we well know, that the loose, spongy structure of the bones of strumous patients as evidenced in the frequent diseases of the bones about the joints, predisposes to the condition of caries whenever local or other causes shall lend their assistance. Of course I speak in a general way, for not every scofulous patient will have caries, while many a strong, robust person may be so affected. Constitutional causes are certainly far more powerful in the production of this disease than are local injuries; for injuries are common, while cases of caries are comparatively rare.

The slowness or rapidity of this disease are greatly influenced by various individual conditions, being, for instance, quite rapid during the process of dentition, as also in mercurially weakened ones it seems almost but a simple mechanical disintegration; while at other times it seems to but a semi-fatty degeneration, the animal portion of the bone becoming quite soft and greasy.

Many contend that tubercle is present in any and all points of the manifestation of caries; but although as I have already said, such a relationship often does exist, as in the girl before us, yet it is not always present.

Now, I do dot think that the disease is very extensive in the case we are now considering, yet it is impossible to determine that question precisely, since there is no abrupt line of demarcation, as in necrosis, but rather a gradual fading or shading out of the diseased into the healthy structure.

Having, then, a case of caries, we proceed to treat it. This one has now passed the acute inflammatory stage, so that cathartics, diaphoretics, leeches, etc., would be only productive of injury. We have therefore, to treat it in its chronicity, and this we shall do by iron, quinia, beef, cod liver oil, etc., together with a local stimulating injection, consisting of tinct. iod. and tinct. capsic. comp. thrown in once in two or three days, which will assist in the rapidity of the cure. This will be our course for a short time, while we carefully watch whether nature be herself competent, with this assistance, to repair the difficulty, but failing in this we shall then cut down through the track of the fistula and remove all the diseased bone. The tooth-roots, the original cause, have already been removed, so that we have now no inconvenience from that source.

In removing the diseased bone we always use the chisel, and with this instrument you will soon acquire the delicacy of touch that will enable you to detect the instant that healthy bone is reached, by its hard, springy character, while carious bone is soft and brittle, and presents to the sight a dark, deadish-white, or oleaginous appearance, instead of being white and vascular.

All the softened bone should be removed, and the hemorrhage will seldom be severe. Simple alum-water is sufficient, as a general thing, for its control, even, indeed, if this is required. Such will be our treatment, and you shall watch the case with me, and thus know the result.

*Case* II.—This old woman, 65 years of age, has been suffering for months from a continued discharge of pus into her mouth and from a tumor in the roof of that cavity, which has been variously diagnosed, and is giving her much alarm. As you look into her mouth you see this tumor projecting from the roof, and occupying nearly the whole of the right side, being of the size of the largest pea-nut shell. The gums are red and swollen, and pus is exuding from about the necks of numerous decayed fangs which occupy the upper jaw. The tumor itself is hard, yet, upon firm pressure, it yields, and I am sure that it contains pus, a fact which I easily make apparent to you by this exploring needle.

This trouble has existed for over a year, and evidently has its history the usual one of alveolar abscess; but this abscess, from some cause, did not break into the mouth, as it should have done, but the pus took a direction inward, along the palatal plate of the superior maxilla, and is now pointing here. It has also done more than work along this bone. It has set up an ostitis, which has been followed by death of the part, and now we have quite extensive necrosis. I pass a bistoury into this tumor, and you see the instant discharge of pus, which is horribly offensive, characteristic in odor of necrosis. The probe shows the bone to be denuded and dead feeling like a piece of hard wood, or like lead.

Now the cause of all this trouble is evidently situated in these old fangs, some six or eight in number, and all of these we shall remove, thus not only ridding ourselves of their further prejudicial effect, but also giving more free exit to the pus.

Having removed all these teeth, we will wait for a little time to allow the turgid, congested condition of the gums and surrounding parts to subside, that we may be the better able to judge of the progress of the case. Yet, I am sure that the dead portion, called a sequestrum, has not yet separated, and that we must wait a little time until nature shall have cast off this foreign mass, which she will do by forming a line of demarcation between the diseased and healthy structures. Must we wait entirely, however, for this slow and tedious process? Yes, we cannot hasten it greatly; yet there is a means which has been proposed by Mr. Pollock in these cases concerning which, however, I have not had sufficient experience to speak with certainty. We will try it in this case, however, and test its merits.

This treatment consists in the injection of sulphuric acid, mixed with six quarts of water, into the parts; the operation to be repeated every day. The acid sulph. arom. may also be used in its full strength. The dressing should consist of tannic acid ℥ ij, glycerin ℥ j, M. Ft. pasta. It is claimed that the cure is expedited, and much more satisfactory results obtained, by thus dissolving out the inorganic constituents of this bone, just as you have seen done by your anatomists.

Now, necrosis of the superior maxillary bones is far less common than that of the inferior; and although it is more liable to take on a general inflammation, yet its higher vascularity and susceptibility enables it to resist the destructive action and limit the part overwhelmed, so that it is seldom affected in its entirety. In this case before us I judge that a large portion of the right palatal plate and of the alveolar process will separate.

Sometimes the necrosis only affects the alveolar border by itself, being in such cases often the result of a local irritant, other than an inflammation of the alveolar-dental periosteum, as for instance from the use of arsenic, chloride of zinc, etc., in destroying the pulp of teeth, such paste has oozed down around the necks of the teeth, and developed a severe inflammation, ending in the death of the process; limited however in extent, and in its duration, four weeks being sufficient for its separation.

The separation of a bone slough in any position is a long and tedious process, requiring patience in the surgeon, and unlimited faith on the part of the patient, especially as during all the time the case may seem—superficially viewed—to be steadily growing worse. It will often be fortunate for your reputation if these cases do not reach you until the sequestrum has been already separated, and is lying cooped up by the tissues—that is provided you cannot have seen them in tne first stage,—since few patients have the resolute de-extermination and abiding faith to resist the constant discouragement of appearances, and friends who will clamor that "something shall be done," and I have usually found it true that those who know the least, advise the most.

We must always pay careful attention to the building up and supporting the constitution of the affected person, or repair and separation will not go on. We will administer the best of tonics and food to this old woman.

When it is seen that death of the body of a bone is certain, especially in the inf. max., it will be found of all advantage to endeavor to have the dying bone replaced by new structure, just as new bone is formed around a necrosed tibia. To accomplish this, I slowly enucleate the periosteum—the bone forming structure—by means of small tents of cotton or sponge introduced between it and the dying bone, which by their expansion slowly strip it away, thus hastening the death of the old part and retaining the valuable periosteum. In this we can sometimes, but not always envelop the old bone with the new growth, and thus prevent deformity. When the periosteum is indisposed to form such new bone, it may be favorably stimulated by tinct. iodine, with a small addition of creosote, especial attention being given at the same time to cleanliness and disinfection. Weak injection of potass. permang., two or three grains to ℥j of water will answer nicely.

Even should these fail and the progress be slow and tedious, do not let your impatience overcome your better judgment, but continue to wait, for I have found from experience that all operative interference in cases of maxillary necrosis is only productive of harm, and especially is this true of phosphor-necrosis, of which I shall speak to you upon some future occasion.

*Case* III.—This woman presents a pouting, red teat-like prominence beneath the base of the lower jaw, with an opening in its centre which is so characteristic that any one of you would at once say that there was dead bone beneath. From this teat is a constant flow of pus, and although my probe passing through the sinus reveals dead bone, yet it is not a detached sequestrum and we must here also wait for the separation of the slough.

It is somewhat singular, notwithstanding the immense amount of material applying at this clinic, that is should so often happen that a number of almost similar cases should be found upon a single day ; yet this is often the case, as you have frequently the occasion to notice both in my service and in that of Prof. Agnew. Upon a previous occasion I showed you a number of cases of necrosis in all of which the sequestra had already separated, and were removed by cutting down upon them either in the line of the sinus, or through the jaws; yet in the cases to-day not one of them is ready for operation.

*Case* IV.—Here is also a case of necrosis of long standing, which is not due to alveolar abscess, as were all the preceeding ones, but to the fracture of the jaw inflicted some five years since. The fragments united, but a portion of the bone died, and was finally removed by operation; yet this did not cure her trouble, for from some unknown cause, portions of bone still continued to necrose, and keep up a constant discharge. Several fragments have been removed at various times, and she presents numerous scars along the base of her jaw, but there is yet bone to come away, and we can only hasten it by the occasional use of the stimulating injection of which I have spoken.

One important part of the treatment, however, we must not neglect. This constant tendency to death of the bone shows that there is a constitutional fault lying behind this disease, and, if I mistake not her appearance, she is tainted with that dire disease, syphilis. Now, shall we dose her with the routinist's iodide of potash and corrosive sublimate? She is pallid, feeble and anæmic to the extreme, and such a course would but still further reduce her already poor blood. No, let us try to give her good red corpuscles, and thus the nutrient material being rich, produce healthy action in the bone and give nature the mastery. I think, then, that the syr. ferri iodid. will be far better for present condition, which, it is alterative, is also tonic in its action. We will commence with ten drops ter die, and increase to fifteen if the stomach is tolerant, and it would not be amiss to add a few drops of liq. potass. arsenitis to each dose.

Of course her hygienic circumstances are unfavorable, yet we will try to place her in the most favorable condition possible, and order as good food as her circumstances will permit. Cod-liver oil will add greatly to her nutrition; in fact, in this weak, feeble state of syphilitic necrosis, I may say that it is a far better "specific" than is potass. iodid. With these measures I trust that we shall be able to limit this unhealthy action, and compel it to be cast off leaving behind a healthy bone.

Case III. will be treated with the same stimulating injection.—*Prof. Garretson's Clinic in Uuiv. of Pa., Reported by Dr. Willard in Med. and Surg. Reporter.*

---

